# Tailoring surface phase transition and magnetic behaviors in BiFeO_3_ via doping engineering

**DOI:** 10.1038/srep09128

**Published:** 2015-03-16

**Authors:** Feng Yan, Guozhong Xing, Rongming Wang, Lin Li

**Affiliations:** 1 School of Engineering and Applied Sciences, Harvard University, Cambridge, MA. 02138, USA; 2 School of Mathematics and Physics, University of Science and Technology Beijing, Beijing 100083, PR China; 3 Department of Metallurgical and Materials Engineering, University of Alabama, Tuscaloosa, AL. 35408

## Abstract

The charge-spin interactions in multiferroic materials (e.g., BiFeO_3_) have attracted enormous attention due to their high potential for next generation information electronics. However, the weak and deficient manipulation of charge-spin coupling notoriously limits their commercial applications. To tailor the spontaneous charge and the spin orientation synergistically in BiFeO_3_ (BFO), in this report, the 3*d* element of Mn doping engineering is employed and unveils the variation of surface phase transition and magnetic behaviors by introducing chemical strain. The spontaneous ferroelectric response and the corresponding domain structures, magnetic behaviors and spin dynamics in Mn-doped BFO ceramics have been investigated systematically. Both the surface phase transition and magnetization were enhanced in BFO via Mn doping. The interaction between the spontaneous polarization charge and magnetic spin reorientation in Mn-doped BFO are discussed in detail. Moreover, our extensive electron paramagnetic resonance (EPR) results demonstrate that the 3*d* dopant plays a paramount role in the surface phase transition, which provides an alternative route to tune the charge-spin interactions in multiferroic materials.

Poise as the most promising materials for the design and construction of multifunctional devices, multiferroics are renowned for their unique and large coupling of electric, magnetic and structural order parameters^1^,^2^. Among most popular multiferroic materials, BiFeO_3_ (BFO) has attracted tremendous interests due to the co-existence of ferroelectricity and ferromagnetism in a single phase^3^. The ferroelectricity of BFO is originated from the 6*s*^2^ lone pair electrons of Bi^3+^ ions structural distortion while the magnetic property comes from the Fe-O-Fe superexchange interactions^4^,^5^,^6^. In addition, BFO features with high ferroelectric Curie temperature of ~1103 K and antiferromagnetic Néel temperature of ~643 K, strong correlations among crystal structure, ferroelectric property and spiral spin structure, and interaction between spontaneous charge and spin. Many research efforts have been placed on manipulating the ferroelectric polarization or the ferromagnetic behaviors using the external filed, such as electric filed to tune the magnetic domain or conversely, using a magnetic field to tune the ferroelectric polarization^7^,^8^,^9^. Introducing a donor-type dopant into BFO, on the other hands, provides an effective intrinsic way to control the ferroelectric domain switching behaviors, and manipulate the spontaneous charge and spin by the chemical strain. A dedicatedly chosen dopant can distort the FeO_6_ octahedra, and modify the concentration of the oxygen vacancies, spin and even surface charge density significantly. Meanwhile, the introduction of dopant modulates the periodicity of spin, which leads to the glassy behaviors^10^. The impact of the structure disorder on the electronic phase transition and magnetic behaviors in doped BFO is still unclear.

In this report, we investigate the effect of Mn doping in BFO on controlling the surface phase transition and magnetic behaviors using electron paramagnetic resonance (EPR) spectroscopy. Furthermore, transmission electron microscopy (TEM) and piezoresponse force microcopy (PFM) techniques are employed to characterize the correlation of microstructures with doped macroscopic/microscopic, ferroelectric domains structures and magnetic properties of Mn-doped BFO specimens. The interactions between charge and spin are discussed in detail to elucidate the unique surface phase transition in Mn doped BFO at low temperature.

## Results

### Synthesis, chemical and physical characterizations

Details on the powders synthesis, chemical and physical characterizations using x-ray diffraction (XRD), transmission electron microscopy (TEM), piezoresponse force microscopy (PFM), superconducting quantum interference device (SQUID) magnetometer, differential scanning calorimetry (DSC) and electron paramagnetic resonance (EPR) spectroscopy can be found in **Methods**. We name the undoped BiFeO_3_, 0.5 at.% Mn and 2.0 at.% Mn doped BiFeO_3_ as BFO, 0.5% Mn-BFO and 2.0% Mn-BFO, respectively.

**Figure 1:**
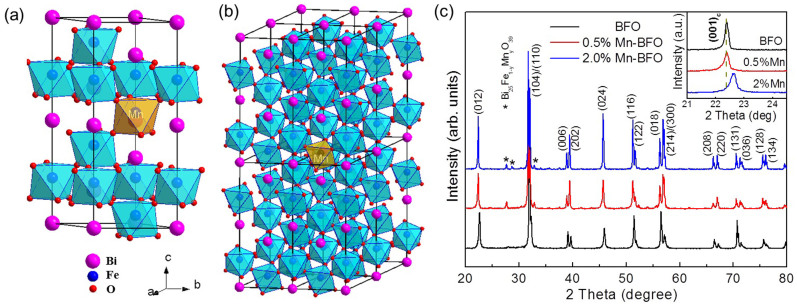
Schematic atomic crystal structures illustration of BFO unit cell with indication of Mn substitution lattice site (a) and 2.0% Mn-doped BFO supercell (b). Bi, Fe, Mn and O atoms are colored in purple, blue, light-yellow and red, respectively. (c) Wide-angle XRD patterns with an inset of magnified (001)_c_ peaks nearby 2*θ* = 22.5°.

Figures 1(a) and 1(b) display the atomic configuration of BFO unit cell with Mn substitution and 2.0% Mn-doped BFO supercell, respectively. Notably, BFO has a rhombohedrally distorted perovskite-type structure of space group *R*3*c*, and its unit cell has the lattice parameter *a* = 5.57874 Å and *c* = 13.8688 Å [Fig. (a)]. The distorted FeO_6_/MnO_6_ octahedra are formed with Fe^3+^ ions surrounded by six neighboring oxygen anions, and two octahedra are connected by sharing their oxygen. The introduction of the Mn ions into the Fe ion site significantly destabilizes the pole symmetry due to chemical strain effects, which stem from the size mismatch between the two B-site cations (Mn and Fe ions)^11^. Additionally, the multi-valence states of Mn ions provide additional force to drive the charge compensation. Figure 1(c) shows the XRD patterns of the undoped and Mn-doped BFO samples with various Mn doping concentrations. All XRD patterns can be identified as a rhombohedral phase (JCPDS File No. 71-2494) with space group of *R*3*c*. Clearly the crystallographic symmetry has not been modified by the Mn substitution, since the Mn solubility in BFO is close to 30% at Fe substitution sites^12^. A small amount of Mn-related secondary phase of Bi_25_Fe_1-y_Mn_y_O_39_ is observed within the detection limits; and yet this phase is a paramagnetic phase so that it will not affect our analysis discussed later^13^. As shown in the inset of Fig. 1(c), the cell parameters calculated from the (001)_c_ peak based on the pseudocubic lattice type are 3.969, 3.955 and 3.941 Å for BFO, 0.5% Mn-BFO and 2.0% Mn-BFO, respectively, confirming that the different ionic radii of Mn and Fe ions generate the chemical strain.

**Figure 2:**
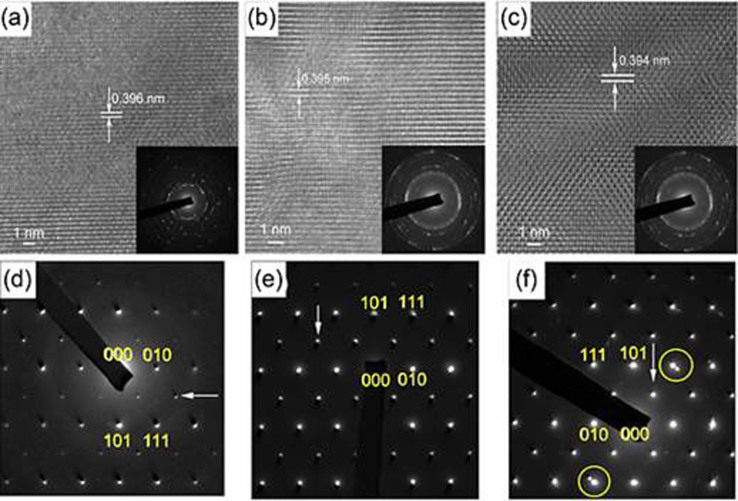
HRTEM images of (a) undoped BFO, (b) 0.5% Mn-BFO and (c) 2% Mn-BFO accompanied with the SAED patterns. Corresponding electron diffraction (ED) patterns from <110>_c_ zone axes in (d), (e) and (f), respectively. Superstructure reflections associated with antiphase rotation of the FeO_6_ octahedra are arrowed and the second phase patterns in 2% Mn-BFO are ringed in yellow color.

Figures 2(a), 2(b), and 2(c) show the high-resolution TEM (HRTEM) images of the undoped and Mn-doped BFO samples, respectively, along with the corresponding selected area electron diffraction patterns (SAED) shown in the insets. These results further confirmed that the BFO samples are well crystallized with perovskite structure, which are consistent with our XRD results. The resolved crystalline domain with a uniform interplanar spacing of 3.96, 3.95 and 3.94 Å are corresponding to the (001)_c_ *d*-spacing of the pseudocubic structure. The electron diffraction (ED) patterns in Figs. 2(d), 2(e), and 2(f) reveal the details of octahedral tilting in the samples^14^. The superstructure patterns are associated with the antiphase rotations of FeO_6_ octahedra at 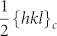 positions around six out of 12 <110>_c_ zone axes^15^. The intensity of the superstructure reflections is enhanced with increasing Mn content, indicating that the FeO_6_ octahedral symmetry has been significantly tilted due to the Mn substitution at the Fe-sites. The tilted octahedral structure will further influence the spin orientation of BFO host as a consequence of the change of “-Fe-O-Fe(Mn)-” bond angle and the perturbation of the spatial spin modulation. Meanwhile, the atomic (polar) displacements and the chemical strain in the bulk surface and inside are not the same, which can result in different charge release^16^.

**Figure 3:**
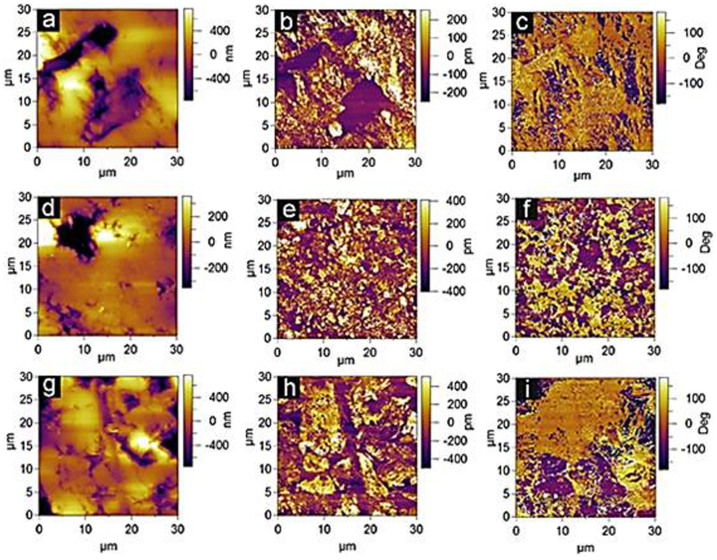
(a), (d) and (g) AFM images (30 × 30 μm^2^), (b), (e) and (h) out-of-plane PFM amplitude images, and (c), (f) and (g) PFM phase images of undoped BFO, 0.5% Mn-BFO and 2% Mn-BFO. Yellow (bright) and purple (dark) on the PFM image correspond to upward (yellow) and downward (purple) domains, respectively.

The piezoresponse force microscope (PFM) is used to evaluate the piezoelectric response of samples^17^,^18^. The surface morphologies of the polished samples are shown in Figs. 3(a), 3(d) and 3(g). The AFM images were recorded in an AC scanning mode with a deflection set point of 0.2 V and at a scanning rate of 0.5 Hz. The spring constant of the tip is about 2 N/m. It is clear that the surfaces are smooth enough for PFM scanning. The PFM experimental setup was described in detail previously^19^. The resolved PFM amplitude images are shown in Figs. 3(b), 3(e) and 3(h). In comparison with the undoped BFO, the Mn-doped BFO samples exhibit an enhanced piezoelectricity response according to the distribution of the piezoresponse amplitude obtained from PFM amplitude histogram. Figures 3(c), 3(f) and 3(i) show the out of plane PFM phase images. The majority of the domains in undoped BFO are oriented with the polarization upward (yellow bright), and only small portion of the domains exhibits the polarization oriented downward (corresponding to a smaller concentration of the 180° domain walls). In contrast, Mn-doped BFO phase images show a ratio between upward and downward polarized domains close to 6:4 for 0.5% Mn-BFO and 4:6 for 2.0% Mn-BFO, respectively. A number of areas in different samples were scanned via PFM to confirm the domain distribution is uniform in the sample. Furthermore, the Mn-doped BFO specimens exhibit a higher volume density of domain walls than that of undoped one, suggesting that the Mn ion can effectively reduce the domain size in BFO^20^. This modification of ferroelectric domain structure via Mn doping enables us to utilize the chemical strain to control and tune the spontaneous charge state. Meanwhile, the electronic transition between the domain and domain wall opens a door for us to incorporate magnetic degree of freedom into multiferroic BFO. Before delving into the magnetic behaviors, the macroscopic ferroelectric and dielectric properties were investigated first at room temperature.

**Figure 4:**
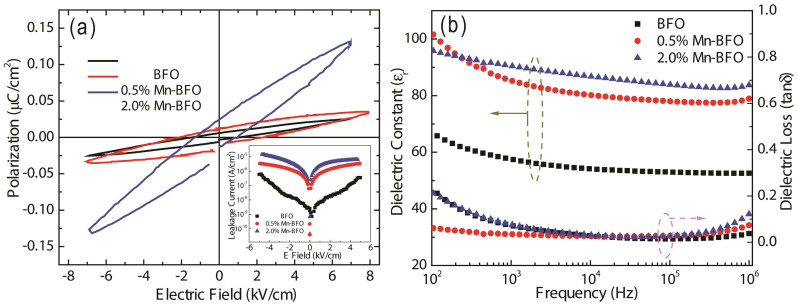
(a) Polarization hysteresis (*P*-*E*) loops, inset shows the leakage current density and (b) variation of the dielectric constant (*ε_r_*) and dissipation factor (tan*δ*) with frequency for undoped BFO, 0.5% Mn-BFO and 2% Mn-BFO.

Figure 4(a) shows the ferroelectric polarization versus electric field (*P-E*) curves of the undoped and Mn-doped BFO samples measured at ~1 kHz frequency and at room temperature. Noted that the loops are not saturated, which can be ascribed to the leakage current due to the defects (e.g. oxygen vacancies), and the relatively low applied electric field cannot switch the ferroelectric domain effectively for thick samples. Nevertheless, with Mn additions, an enhancement in polarization is achieved. The enhanced polarization can be attributed to the elevated density of ferroelectric domains, considering that the domain structure obtained via PFM in Mn doped BFO sample, Additionally, the Mn substitutions increase the distortions in the FeO_6_ octahedral and Fe-O-Fe bond angles, and thus the tetragonality in the crystal structure. The resultant chemical strain from the structure variation can augment the polar displacement of Bi^3+^ ions and the 6*s*^2^ lone pair electrons of Bi^3+^ ion^21^, as a result, the increased polarization is as expected. The leakage current density (*J*) versus the applied electric field (*E*) is shown in the inset of Fig. 4(a). Undoped BFO shows the lowest leakage current, while the leakage of Mn-doped BFO samples are nearly one order of magnitude higher than that of undoped BFO. This is consistent with the increased density of more conductive ferroelectric domain walls in Mn-doped BFO. As for the Mn-doped BFO, the leakage current increases with increasing Mn concentration. This can be ascribed to the fact that the substitution of Mn on the host Fe site provide more electrons due to Mn ion multivalent compared with Fe ions, which agrees with the previous results^22^.

Figure 4(b) illustrates the dielectric constant, *ε_r_*, and dielectric loss, tan*δ*, as a function of frequency for pure and Mn-doped BFO. At frequency (10^2^ ~ 10^6^ Hz), *ε_r_* and tan*δ* of the samples varies slightly. The Mn doping significantly enhance the dielectric constant, whereas only limited influence is noticeable on the dielectric loss. This is due to the charge compensation of the charged defects, such as 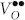, 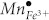 or defect complex 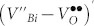. At high frequency (>10^5^ Hz), a slight increase of tan*δ* is observed, indicating that the charge carriers cannot catch up with the frequency of the applied field. It is noteworthy that the increase of dielectric loss at low frequency is highly likely originated from the space charges effects^23^.

**Figure 5:**
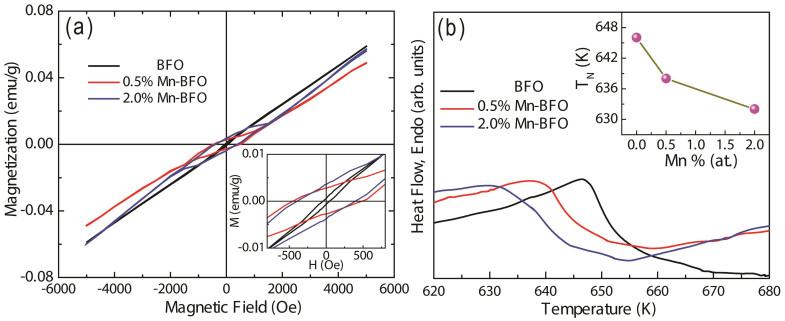
(a) Field dependence of magnetization, the inset shows the magnified *M*-*H* curves. (b) DSC signal *vs*. temperature traces for undoped BFO, 0.5% Mn-BFO and 2% Mn-BFO, the inset shows the Néel temperature of BFO as a function of Mn doping contents.

Magnetization versus magnetic field (*M*-*H*) curves of all samples are measured at a maximum magnetic field of 5000 Oe at 300 K as shown in Fig. 5(a). The *M*-*H* hysteresis loops [inset of Fig. 5(a)] clearly verify the ferromagnetic nature. The enhanced magnetization is ascribed to the fact that the Mn ions change the canting of antiferromagnetically ordered spins^24^. The net magnetization might increase with the variation in canting angle^25^. In addition, Mn dopants can break the superexchange between Fe ions. According to the Goodengough-Kanamori rules^26^, the superexchanges of Fe^3+^-O-Fe^3+^ and Fe^3+^-O-Mn^3+^ are antiferromagnetic, while the superexchanges of Fe^3+^-O-Fe^3+^ and Fe^3+^-O-Mn^4+^ are ferromagnetic. The increase in the Mn content results in less antiferromagnetic superexchange interactions but induces more frustrated antiferromagnetic superexchange interaction^12^. This reduces the Néel temperature, *T*_N_ and enhances macroscopic magnetization, which is in good agreement with our DSC measurements. The DSC curves for undoped and Mn-doped BFO are displayed in Fig. 5(b). The inset of Fig. 5(b) shows the Néel temperature (*T*_N_), which is determined by the specific heat measurement, as a function of Mn-concentration. The *T*_N_ value decreases with increasing Mn content at a rate of 0.63 K per 1% mole Mn, which is calculated through a linear fitting of the experimental data. Then we apply a simple rule of mixture and calculate *T*_N_ as the weighted average between the transition temperature of BFO and BiMnO_3_,
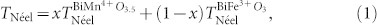
where *x* is the molar concentration of Mn in the mixture. Given that 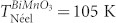
27 and 
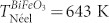
, the estimated decreasing rate of *T*_N_ is 0.54 K per 1% mole Mn, which is smaller than that obtained from our experimental results. Such discrepancy can be attributed to the oxygen vacancies (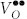) which act as the charge compensation associated with the reduction of Mn^4+^ to Mn^3+^ ion states^28^.

**Figure 6:**
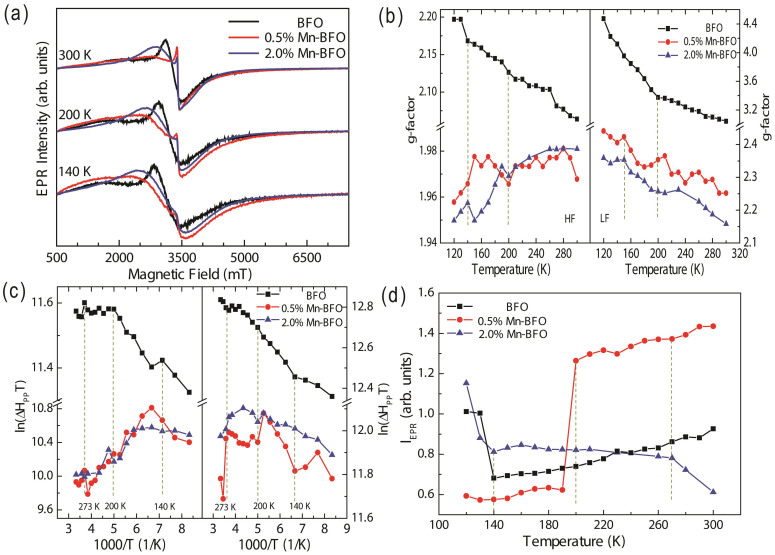
(a) Electron paramagnetic resonance measurements of undoped BFO, 0.5% Mn-BFO and 2% Mn-BFO at various temperatures. Temperature dependence of the *g*-factor (b), the temperature dependence of peak-to-peak linewidth, ln(Δ*H_pp_T*) (c). (d) Integrated intensity *I_EPR_* in the 120 to 300 K illustrating the anomalies. Dash lines are used to guide for the eyes.

To determine the interaction between the electronic properties and the magnetic spin behaviors, we perform a series of measurements of EPR spectroscopy. These measurements are very sensitive to the surface phase transition involving the electronic charges and magnetic spin dynamics^16^. Here, the *X*-band EPR spectra were measured from 120 to 300 K for Mn-doped BFO. As shown in Fig. 6(a), Mn doping remarkably affects the EPR spectra. A typical ferromagnetic (FM) spin-wave resonance can be divided into two resonances segments, i.e., low field (LF) and high field (HF) resonance shoulders, which are related with distinct defect types and magnetic anisotropy. The LF resonance with *g*-factor near 4 has a characteristic of magnetically isolated high spin Fe^3+^ (*S* = 5/2) in a low symmetric environment, which corresponds to the defect complexes, e.g., 
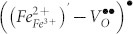
 defect dipoles. On the other hands, the HF resonance with *g*-factor close to 2 can be ascribed to the Fe ions and is correlated with the resonant absorption in the cycloidal spin structure and the defect induced free spins^29^,^30^. Furthermore, a striking absorption splits into two peaks in the Mn-doped BFO spectra, indicating that the magnetic environment for the unpaired electrons in Fe ions has significantly changed due to the Mn ion substitution and the existence of Fe^3+^ -exchange-coupled magnetic secondary phase^31^,^32^.

The theoretical g factor can be expressed in terms of spin Hamiltonian^33^,^34^
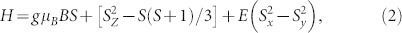
where the first term describes the electronic Zeeman interaction with *g* = 2.0023, *B* and *E* are the axial and rhombic zero-field splitting (ZFS) parameters, *S_x,_ S_y_* and *S_z_* are components of spin along three mutually perpendicular axes *x*, *y* and *z*. In a polycrystalline sample, the axis of symmetry of the different magnetic centers is randomly oriented with respect to the magnetic field, therefore, only one resonance line corresponding to the higher field with an isotropic *g*-factor value ~ 4 can be observed^35^. The *g*-factor value exhibits a sharp increase at the HF resonance whilst it decreases at the LF resonance with increasing temperature, as shown in Fig. 6(b). The *g*-factor value is strongly correlated with the Mn concentration at higher temperature in LF resonance range, suggesting the existence of a high concentration of 
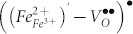
 and Fe defect dipoles in a lower Mn concentration doped BFO. A sharp variation at HF below 190 K can be attributed to magnetic fluctuations as the establishment of long range order^36^. The spin-reorientation stems from the charge localization at low temperature and thermally activated hopping-induced ferromagnetic interactions^37^. The surface phase transition at 140 K and spin-glass behaviors at 200 K were reported for BFO, which is due to interaction between the structural strain (i.e., the atomic displacements and oxygen octahedral tilts) and the spin^16^. Our results also reveal these anomalies in pure BFO, as shown in the Fig. 6(b), where *g*-factor for BFO shows transitions at 140 and 200 K in HF and LF, respectively. In addition, the transition at ~ 270 K is ascribed to the polar H_2_O molecules^38^,^39^ melting from the left condensed water during cooling, which is beyond scope of the present work. The surface phase transitions were also observed in Mn doped-BFO samples, reflecting that the Mn doping can tailor the charge release and the onset of glassy behaviors. Clearly, with increasing Mn ion concentration, the phase transition occurs at higher temperatures, indicating that the more the Mn ions the more energy is required to drive the transition. Moreover, the degree of canting of spins is also related with the *g*-value and a large *g* value results in a severe spin canting. With increasing Mn ion concentration, the short-range antiferromagnetic superexchange interaction transfers to a long-range antiferromagnetic coupling due to the distorted crystal structure^35^.

The linewidth is related with the spin-spin relaxation time and the spin-phonon relaxation time^34^. The peak to peak linewidth, Δ*H_pp_* is extracted in order to clearly distinguish the temperature dependence linewidth, in particular, the ln(Δ*H_pp_T*) is plotted as a function of 1/T, as shown in Fig. 6(c). The linewidth for the Mn doped BFO is narrower than that of the pure BFO in the temperature range, suggesting that the spin-lattice interaction is larger in Mn doped BFO than that of the pure BFO. The narrowing of the EPR signals can be ascribed to the hopping of the *e*_g_ electrons, which averages out the spin-spin interactions between Mn and Fe magnetic moments, such as Dzyloshinsky Moriya (DM) exchange interaction and the anisotropic crystal-field (CF)^40^. The increased spin-lattice interaction in Mn-doped BFO can contribute to the charge injection during the phase transition at 150 K. Figure 6(d) shows the integrated intensity *I_EPR_*as a function of temperature, where the integrated intensity of EPR spectra are proportional to the concentration of unpaired electrons in the samples. It is found that the *I_EPR_*for BFO and 2% Mn-BFO decreases abruptly at 140 K, implying that the existence of internal fields caused by charge release. This behavior is associated with the long-range antiferromagnetic superexchange interaction between Fe-O-Fe for BFO and Fe-O-Mn for 2.0% Mn-BFO, respectively. In contrast, for 0.5% Mn-BFO, an abrupt increase occurs at about 200 K, which is corresponding to the spin rearrangement. The long range antiferromagnetic ordering of magnetic moments does not happen at low temperature. As temperature increases to 200 K, the long-range antiferromagnetic super exchange interaction between Fe-O-Mn commences. The Mn ion EPR signal is contributed by both the tetragonal Jahn-Teller (*J*-*T*) distorted Mn^3+^ (3*d*^4^, *S* = 2) and Mn^4+^ (3*d*^3^, *S* = 3/2) ions.

## Discussion

Bismuth ferrite, i.e., BiFeO_3_ (BFO) has become an extremely exciting material system nowadays due to its unique properties of possessing room-temperature ferroelectric and magnetic order. Up to now, despite the intense research activities, however, there remain a number of open questions concerning the structure, phase diagrams, ferroelectric and magnetic characteristics in BFO. Doping engineering is widely used to tailor the band structures of bulk and nanoscale materials, promising and facilitating the construction of various multifunctional materials and devices^41^,^42^,^43^,^44^,^45^,^46^,^47^,^48^,^49^,^50^,^51^. To stabilize the perovskite state and to induce ferromagnetism at room temperature in BFO, 3*d* element doping engineering was adopted as an effective strategy in our experiments. This report reveals that, substituting Fe site with Mn ions in BFO induces ferroelectric domain structure modulation, surface phase transition and manipulated magnetic behaviors. Homogeneous samples from microstructural point of view were obtained for all the compositions analyzed. The spontaneous ferroelectric response and corresponding domain structures, magnetic behaviors and spin dynamics in Mn-doped BFO have been investigated systematically. Both the surface phase transition and magnetization are boosted in BFO via Mn doping engineering. The interaction between the spontaneous polarization charge and magnetic spin in Mn-doped BFO are discussed in detail. Our temperature dependent EPR results further elucidate that the 3*d* dopant engineering plays a paramount important role in the surface phase transition and provides an alternative dimension to tune the spin-charge and spin-lattice interactions in multiferroic materials. The extrinsic properties are impossible to be satisfactorily controlled by normal ceramics processing. By tailoring the extrinsic contributions to the dielectric and magnetic properties, the ceramic BFO system might be a valuable multiferroic materials for magnetoelectric/magnetoelastic applications, in particular at room temperature.

In summary, the Mn dopants effect on the ferroelectric domain structure and magnetic phase transitions has been systematically investigated via PFM and EPR. The magnetic phase transitions associated with the spin reorientation are observed in the Mn-doped BFO and the transition temperatures are increased by the substitution of Mn in Fe-site of the BFO. In addition, the electrical conductivity is increased significantly through the charge compensation of electrons and variation of the density of domain walls in the Mn-doped BFO.

## Methods

### Materials synthesis

The pure and Mn doped BFO powders are fabricated using the conventional solid solution methods with starting materials at high purity Bi_2_O_3_, Fe_2_O_3_ and MnO_2_ (Sigma Aldrich, >99.99%) at 830°C for 2 hours. The starting materials are Bi_2_O_3_, Fe_2_O_3_ and MnO_2_ (Sigma Aldrich, 99.99%) powders. These are mixed in an agate mortar and pestle for ~30 min, with acetone added periodically to form a paste. Pellets are pressed uniaxially and placed on sacrificial powder of the same composition in alumina boats. Initial firing was at 830°C for 30 min after which the pellets were ground, repressed, fired again at 830°C for 30 min, ground, and repressed isostatically at 300 MPa, given a final firing at 830°C for 2 hours in air, and then cooled naturally to the room temperature.

### Characterization and instrumentation

The crystalline structure of the BFO powders was characterized using X-ray diffractometer (Shimadzu XRD-7000) with a Cu Ka radiation. Differential scanning calorimetry (DSC) measurements were performed in a Netzsch DSC404C (Selb, Germany) from room temperature to 850°C with heating/cooling rates 10°C min^−1^. TEM was performed by Philips CM200 FEG TEM operated at 200 kV and a FEI Nova G2 TEM operated at 200 kV. TEM specimens were prepared using an FEI Nova 200 Nanolab focused ion beam (FIB) and Zeiss Auriga focused ion beam (FIB). This was done followed by a “lift-out” technique. Magnetization measurements were carried out using a superconducting quantum interference device (SQUID) magnetometer (Quantum Design). The surface topographies, behaviors of polarization, and nanoscale ferroelectric polarization switching of the pure and Mn-doped BFO pellets were characterized using a commercial atomic force microscope (AFM) (Asylum Research MFP-3D) with combination techniques of piezoresponse force microscope (PFM) for the local polarization detection. A Pt-coated cantilever (Olympus AC240, nominal spring constant ~2 N/m, resonant frequency ~70 kHz) was used with a scanning rate of 0.5 Hz. Both surfaces of the sample pellets were polished and coated with silver paste as electrode in order to measure the electrical properties. The macroscopic current-voltage characteristics and ferroelectric measurements were performed using an electrometer (Keithley 6487) and ferroelectric tester (Radian, USA). The dielectric properties were measured at room temperature using an impedance analyzer (Wayne Kerr Electronics 6500B Series) at zero bias voltage over the frequency range from 10^2^ to 10^6^ Hz. The X-band (9.5 GHz) Electron Paramagnetic Response (EPR) measurements were performed on EPR spectrometer with a flow nitrogen cryostat (120 ~ 300 K), and as a standard field marker, DPPH with a *g* value equal to 2.0036 were used for the determination of the resonance magnetic field values.

## Author Contributions

All authors conceived the idea for the work and designed the experiments. F.Y. and G.Z.X. synthesized the samples and performed the chemical, physical, ferroelectric and magnetic properties characterization. L.L. and R.M.W. contributed invaluable suggestions during the entire project. All authors contributed to the analysis and interpretation of the results and the writing of the manuscript.
